# Equity in health care financing: The case of Malaysia

**DOI:** 10.1186/1475-9276-7-15

**Published:** 2008-06-09

**Authors:** Chai Ping Yu, David K Whynes, Tracey H Sach

**Affiliations:** 1 Health Economics Research Group, Brunel University, Uxbridge, Middlesex, UB8 3PH, UK; 2 School of Economics, University of Nottingham, University Park, Nottingham NG7 2RD, UK; 3 School of Chemical Sciences and Pharmacy, and Health Economics Group, University of East Anglia, Norwich, Norfolk NR4 7TJ, UK

## Abstract

**Background:**

Equitable financing is a key objective of health care systems. Its importance is evidenced in policy documents, policy statements, the work of health economists and policy analysts. The conventional categorisations of finance sources for health care are taxation, social health insurance, private health insurance and out-of-pocket payments. There are nonetheless increasing variations in the finance sources used to fund health care. An understanding of the equity implications would help policy makers in achieving equitable financing.

**Objective:**

The primary purpose of this paper was to comprehensively assess the equity of health care financing in Malaysia, which represents a new country context for the quantitative techniques used. The paper evaluated each of the five financing sources (direct taxes, indirect taxes, contributions to Employee Provident Fund and Social Security Organization, private insurance and out-of-pocket payments) independently, and subsequently by combined the financing sources to evaluate the whole financing system.

**Methods:**

Cross-sectional analyses were performed on the Household Expenditure Survey Malaysia 1998/99, using Stata statistical software package. In order to assess inequality, progressivity of each finance sources and the whole financing system was measured by Kakwani's progressivity index.

**Results:**

Results showed that Malaysia's predominantly tax-financed system was slightly progressive with a Kakwani's progressivity index of 0.186. The net progressive effect was produced by four progressive finance sources (in the decreasing order of direct taxes, private insurance premiums, out-of-pocket payments, contributions to EPF and SOCSO) and a regressive finance source (indirect taxes).

**Conclusion:**

Malaysia's two tier health system, of a heavily subsidised public sector and a user charged private sector, has produced a progressive health financing system. The case of Malaysia exemplifies that policy makers can gain an in depth understanding of the equity impact, in order to help shape health financing strategies for the nation.

## 1. Background

The financing of health care is a subject of major concern throughout the world. Health care financing is the activity of raising or collecting revenue to pay for the operation of a health care system [1]. The conventional categorisations of finance sources for health care are taxation, social health insurance, private health insurance and out-of-pocket payments. There are nonetheless increasing variations in the finance sources used to fund health care. Differences in social health insurance are: implementation either at the national or community level, eligibility either on a mandatory or voluntary basis, and contribution either by the individual or the employer. Variations in out-of-pocket payments are its formality (or informality) and function either as co-payment, co-insurance or full cost. Hence the emergence of finance sources such as community health insurance and informal payment.

Policy makers are considering or implementing various financing strategies in order to strengthen health care financing. Such implementation is likely to have a substantial impact on the equity of health care financing. Equity involves a value judgment of fairness on the (accepted magnitude of) variations from the (expected degree of) equality in the population. Equity in health care financing is assessed by the degree of inequality in paying for health care between households of unequal Ability To Pay (ATP) [2]. It is usually represented by the extent to which health care is financed according to ATP.

Equitable financing is a key objective of health care systems. Its importance is evident in policy documents, policy statements, the work of health economists and policy analysts. The commitment to equitable financing is expressed in the policy statements by linking finance to: ATP in Denmark and the UK; equity in Ireland, Portugal and Spain; solidarity in Italy and the Netherlands; and tax financing in Switzerland [2]. Furthermore, the accordance of health payments to ATP is regarded as an important objective in the finance of health care in Belgium, France, Germany, Ireland, the Netherlands, Spain and the UK [3,4]. Policy makers in various countries are seen to commit towards financing health care according to ATP. The Ministry of Health (MOH) in Malaysia subscribes to this commitment by proposing that the nation's contribution to the new national health financing scheme be related to ATP [5].

This paper presents an equity assessment of the health financing system, and draws together all finance sources in Malaysia to evaluate the whole financing system. The second section presents the Malaysian health care system. The third section summarizes the methodology regarding the data, variables and analysis on all Malaysian finance sources and the system. The fourth section presents the empirical results on progressivity for the finance sources and financing system. The fifth section discusses the progressivity, methodological concerns, comparisons with other studies and the policy implications. The sixth section draws the conclusions.

## 2. Malaysian Health Care System

The fundamental principle of the Malaysian health care system is that accessibility to health care not to be related to ATP, particularly in the event of sickness [6]. The government is concerned with the performance of the health care system, whose primary purpose is to improve health of the nation [7]. This stems from the understanding that health represents the human capital, which is the central thrust to sustainable economic growth and development of the country. The Malaysian health care system has been improving over time, such that a higher standard of health status has been achieved with the relatively limited resources available to the health sector. For example, throughout the period of 1990 to 2005, life expectancy at birth increased significantly (males from 69.0 years to 71.8 years, females from 73.5 years to 76.2 years), the infant mortality rate has fallen (from 13.5 to 5.1 per 1,000 live births), whilst maternal mortality rate has been held steady (at 30 per 100,000 live births) [5]. Such improvement in health status has been achieved within the range of 2.0% to 4.0% of GDP being spent on health services in Malaysia. The total health expenditure was 3.1% of GDP during the HES 1998/99 (with per capita total expenditure on health at 112 US$ average exchange rate or 261 international dollar rate), and has increased to 3.8% of GDP in 2003 (with per capita total expenditure on health at 163 US$ average exchange rate or 374 international dollar rate) [8]. Malaysia was ranked at 49 from 191 WHO member countries in the World Health Report 2000 [9], which assessed the overall health system performance against three objectives of good health, responsiveness and fair financial contribution. Malaysia performed unsatisfactory in fair financial contribution, with ranking at 122–123 from 191 WHO member countries, whilst moderately in the other two objectives (the level of good health was ranked at 89 whilst distribution at 49 from 191, the level of responsiveness was ranked at 31 whilst distribution at 62).

### 2.1 Delivery of Health Care

A dual health care system, with both the public and private health services, co-exists in Malaysia. The government provides health care services to the nation through public hospitals and health clinics throughout the country. The services range from outpatient curative care to preventive and promotion of health. The main public health provider is MOH that provides primary care, secondary care and tertiary care through various types of health facilities (such as general hospitals, district hospitals and health clinics). There were 122 MOH hospitals (with a total of 30,021 beds), 6 special medical institutions (with 4,740 beds), 809 health clinics, 1,919 rural clinics, 89 maternal and child health clinics, and 146 mobile clinics in 2005. An open-door policy in regard to general outpatient services and hospital admissions has been practiced by the public health sector. Access to specialist services is nonetheless controlled through a national system of referral. Specialist services are available at designated hospitals (such as national referral hospital in the capital, the state hospital and selected district hospitals). Referral of patients for specialist services is to the nearest facility if patients cannot be managed at general outpatient facilities. The National Quality Assurance Programme was implemented to maintain, improve and evaluate the quality, efficiency and effectiveness in the delivery of public health services [7]. The Clients Charter commits providers to providing a specified standard of services explicitly and can be used in order to monitor the quality of services and enhance customer satisfaction.

Public health services are heavily subsidized by the government. Primary care services at health clinics are delivered almost free of charge, whereby each patient is charged a nominal fee of RM 1 (equivalent to US$0.31 in 2007) for each outpatient visit based on Fees (Medical) Order 1976. Secondary and tertiary care services provided at hospital facilities are also highly subsidized by the government. A total of RM 7.8 billion (equivalent to US$2.4 billion) was allocated to the MOH for funding the public health services in 2005 [8]. The fees collected by the MOH nonetheless only constitute about 2% of the MOH budget in 2004, which means that the Government subsidized about 98% of the health services provided by the MOH [10].

Private health providers complement the medical services provided by the government. Private health providers mainly focused on curative services and include general practitioner clinics, medical centres to private hospitals [7]. Private hospitals exist in a variety of sizes (with the number of beds ranging from 17 to 2,358). There were 218 private hospitals (with a total of 10,542 beds), and an estimate of about 5,000 private general practitioner clinics (providing a range of primary health services) in 2004 [11].

The emergence of a private health sector is driven by demand. Affluent members of the population expect high quality health services and create the demand for a private sector. The quality of care at private facilities was perceived to be of high quality [7]. Newly built private hospitals are equipped with large, ultramodern and lavish medical technology. The prompt services at the private general practitioners' clinics also offer convenient medical services in particular to the nearby population [7].

The private facilities are monitored and regulated by the Malaysian government to ensure quality service and cost control. The regulatory environment was strengthened by the implementation of Private Health Care Facilities and Services Act 1998 enforced on the private sector. It expresses specific requirements for facility standards and the assurance of quality services in accordance with the National Quality Assurance Programme [5].

The private sector charges user fees on patients for utilizing health services in order to operate and maintain their facilities. The private sector offers lucrative remuneration packages to medical practitioners upon joining their organization. The expense of utilizing health services at private facilities (represented by their user fees) is higher than at public facilities. Access to private health services is inevitably limited to the richer segments of the population that can afford to pay high user fees as out-of pocket payments or co-payments (with coverage of private insurance)[7].

Some private hospitals are established as charitable institutions, in parallel with the principles of a caring society. This principle is one of the nine challenges in the Vision 2020 [7], which articulated the government's vision of a developed and united nation. Price discrimination is practiced in some of the charitable institutions, by charging a premium on those who can afford for cross-subsidization to the poor. The price discrimination practice is nonetheless becoming increasingly difficult to sustain due to the need to compete with commercial hospitals [12].

### 2.2 Financing of Health Care

Malaysia is a predominant tax financed system that the government contributes significantly towards financing health services. The national (macro) level expenditure showed that the government subsidise 58.2% of the funding in public health sector whilst the balance of 41.8% is financed by the private sector in 2003 [8]. A tiny 0.8% of general government revenue consisted of social security contributions. The majority of private finance sources were accounted for by out-of-pocket payments (73.8%) with a minor component of private insurance (13.7%). The balance 12.5% of private finance sources is unreported in the WHO 2006 [9].

**Figure 1:**
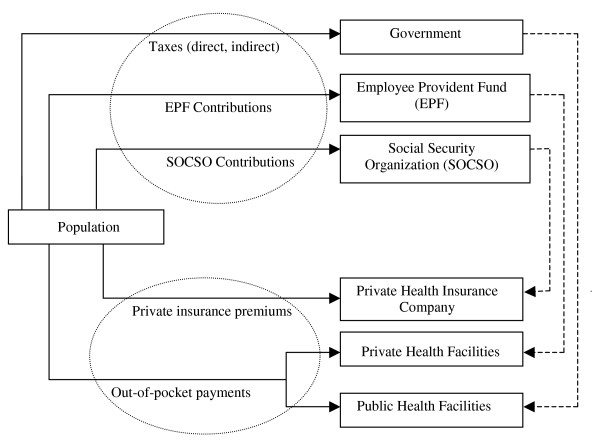
Households' Financial Contributions to Health Care System in Malaysia.

Malaysian health services are ultimately funded through the general population by tax payments, and contributions to EPF (Employee Provident Fund) and SOCSO (Social Security Organization). The Ministry of Finance (MOF) collects general taxes (as direct and indirect taxes) to finance the public services including health care. The employed population also contributes to EPF. The primary purpose of EPF is to create savings for old age for the contributor and his family, nevertheless, 30% of the individual's contributions can be withdrawn for reimbursement of health care expenditure [13]. The employed population earning less than RM 3,000 further contributes to SOCSO that provides medical benefits for work-related injuries of members [14]. Private insurance is voluntarily purchased by individuals, who pay different premiums depending on the type of health insurance and level of coverage. Out-of-pocket expenses represent payments incurred at the point of utilization at health facilities. The households' financial contributions to the health care system in Malaysia are shown in Figure 1. The five sources of funding (direct taxes, indirect taxes, EPF contributions and SOCSO contributions, private insurance premiums and out-of-pocket payments) are channelled directly or indirectly through financial intermediaries to either public or private health facilities that coexist in parallel.

The utilisation of public health services is almost free, only nominal charges are levied upon certain services whereby the patients have to pay from out-of-pocket. Conversely, utilising private health services require out-of-pocket payments, or co-payments with private health insurance coverage. The affluent or those that can afford to pay user fees have a choice of switching to the private sector whilst the poor rely on the public sector. Private health services were perceived as of higher quality with reduced waiting time [7].

## 3. Methodology

The assessment on equity in financing health care draws on established techniques from the public finance literature. The starting point of assessment is the notion that health care financed according to ATP is considered equitable. To judge whether the health payment undermines or contributes to the equitable financing goal, one has to assess how closely in practice health payment is linked to ATP.

Progressivity measures the deviation from proportionality in the relationship between health payment and ATP [15]. It reveals the extent of inequality in paying for health care services between households of unequal ATP. A health payment is progressive (regressive) if it accounts for an increasing (decreasing) proportion of ATP as ATP rises. A progressive (regressive) system means that the individuals or households with greater ATP are paying more (less) proportionally in financing health care. Health care financing systems are proportional if individuals or households with different ATP are spending the same proportion of ATP in financing health care.

The Kakwani's progressivity index [16], widely used in public finance, is a frequent tool of assessment for equitable financing. It is defined as *η*_*k *_= *C *- *G*, *C *is the concentration index for payments and *G *is the Gini coefficient for income (or consumption, expenditure) [16]. *C *equals -1 if the entire financial burden is concentrated in the hands of the poorest person, and 1 if the financial burden is concentrated in the hands of the richest person. G equals 0 where there is perfect income equality (every individual has the same income), and 1 where there is perfect income inequality (one individual has all the income whilst every other individual has zero income).

*η*_*k *_is positive in a progressive system and *η*_*k *_is negative in a regressive system. The value of *η*_*k *_ranges from -2 (= -1-*G*) in the most regressive system to +1 (= 1-*G*) in the most progressive system. Kakwani's progressivity index at 0 means that the system is proportional and health payments account for the same proportion of income, irrespective of the individuals' income [17].

The Kakwani index was computed directly in a single step from convenient regression in the following form [15]:

$$2\sigma_{R}^{2}[\frac{h}{\eta }-\frac{y}{\mu }]=\alpha +\beta R+\mu$$

where *σ*_*R*_^2 ^is the sample variance of the fractional rank variable, *h *is the health payment variable, η is the health payment variable's mean, *y *is the ATP variable, *μ *is the ATP variable's mean, and *R *is the household fractional rank in the ATP distribution. The Ordinary Least Squares estimate of *β *is the Kakwani index.

### 3.1 Household Expenditure Survey Malaysia 1998/99

This research employed data from the Household Expenditure Survey (HES) Malaysia 1998/99. The HES is a nationally representative survey conducted by the Department of Statistics Malaysia every five years, which aims to collect information on the levels and patterns of consumption expenditure by selected households for a comprehensive range of goods and services [18]. Although the survey's primary purpose is to capture household expenditure patterns, the cardinal measures collected have the potential for equity analysis in health care financing.

The HES 1998/99 was carried out for a period of 12 months, from July 1998 to June 1999. In the survey, a household is defined as a person or group of persons that stays in the selected dwellings (or 'living quarters') for at least 16 nights during the reference month. The selected households were asked to record the daily purchase of all individual items for a month, they were also interviewed individually [18]. A sample size of 9,198 households was employed in this research.

Two crucial variables for this research are ATP and health payments. Consumption was adopted as the preferred measure of ATP for Malaysia, as a developing country where living standards are contributed substantially from the household production [19]. The World Bank [15] assured that consumption or even expenditure is regarded as a better measure of ATP than income in the case of developing countries. The conceptual and practical considerations are firstly, consumption is smooth over time whilst income fluctuates. Secondly, informal labour markets and home production are common and widespread in developing countries, therefore many households may have multiple, variable and changing sources of income [15]. The sensitivity of the Kakwani index towards other proxies of ATP measures, such as gross income and non food consumption are illustrated in the empirical results.

The five finance sources in the Malaysian health financing system are direct taxes, indirect taxes, contributions to EPF and SOCSO, private insurance premiums and out-of-pocket payments. Out-of-pocket payments and private insurance premiums are reported in the HES Malaysia 1998/99. Direct taxes and contributions to EPF and SOCSO are derived whilst indirect taxes are estimated from the expenditure variables in the HES Malaysia 1998/99. The basis of derivation of these five health payments are summarised in Table 1.

**Table 1:** The basis of derivation of Health Payment Measures

Health payment measures	Basis of derivation from the survey
Direct taxes	Represented by expenditure on income tax.
Indirect taxes	Estimated expenditure on sales taxes. Sales taxes estimated from household expenditure (on rates ranging from 5% to 15% depending on the types of goods).
Contributions to EPF and SOCSO	Represented by the summation of employee's and employer's contributions to EPF, SOCSO and others.
Private insurance premiums	Reported expenditure on accident and health insurance premiums.
Out-of-pocket payments	Reported expenditure on pharmaceutical products, therapeutic appliances and equipments, medical and dental services, and hospital services and treatments.

Macro weights of these five health payments are required to assess the overall progressivity of the whole health financing system. Macro-weights established from the MNHA 1997–2002 [20] were used for the main analysis (shown in Appendix 1), as recommended by the World Bank [15].

### 3.2. Adjustment for the Composition of Households

To obtain adult equivalent estimates for progressivity analysis, it is necessary to adjust the household estimates of aggregate expenditure (in the HES data) to reflect household size and composition [15]. Additionally, the HES data should also be adjusted for economies of scale that arise from sharing public goods in the households. These two adjustments can be conducted by applying an equivalence scale. The number of adult equivalents (AE) in the household was defined as:

AE = (A+αK)^*θ*^

where A is the number of adults in the household, K is the number of children, α is the cost of children, and θ is the degree of economies of scale [15].

Deaton and Zaidi [21] proposed a value between 0.3 to 0.5 for α and near unity for θ in developing countries. A higher α represents an increased cost of children in developed countries, whilst a higher θ represents an increased proportion of private consumption (such as food) in the household. Nonetheless, the procedure of identifying equivalence scale has been difficult and arbitrary [21]. Furthermore, an equivalence scale has not been applied in analyzing the HES by the DOS in Malaysia. For the purpose of this research, an adult equivalence scale of 0.5 was used to represent the developing state of an upper middle income country such as Malaysia, in parallel with the study on Asian countries by O'Donnell et al 2005 [22]. An economies of scale at 1 is used to represent the equal proportion of private consumption for each adult in the household (which represent a neutral state without economies and diseconomies of scale). The implication of using different adult equivalent scale and different economies of scale was examined in sensitivity analyses.

### 3.3 Progressivity Analysis

Kakwani's index was first calculated for each five finance sources. The progressivity of the whole financing system was subsequently established by weighting the progressivity (using the macro-weights) of the five finance sources. In order to test the sensitivity of Kakwani's index towards the choice of ATP measures and the application of equivalent scales, sensitivity analyses were conducted using three scenarios. First, per household amounts for payments and consumption (instead of per adult equivalent amounts for payments and consumption with the application of equivalence scale) were used to calculate Kakwani's index. Second, income (instead of consumption) was used as the proxy of ATP. Third, non food consumption (instead of consumption) was employed as the proxy of ATP. Additionally, sensitivity analysis using three different incidence assumptions were conducted for three scenarios (details in Section 4.1).

## 4. Empirical Results

The net progressivity of the five finance sources in the whole health financing system, assessed by Kakwani's indices, is shown in Table 2. Kakwani's indices were obtained directly from the convenient regressions. The Kakwani's index is interpreted in terms of its directions (positive or negative), its quantum, and its significance. The positive sign of four Kakwani's indices reveal the progressivity of all finance sources except indirect taxes. The largest positive value using the Kakwani's index showed that direct taxes were the most progressive, followed by private insurance premiums and out-of-pocket payments. The lowest positive value of contributions to EPF and SOCSO showed that they were the least progressive. The significance of progressivity (or regressivity) is revealed for almost all finance sources except private insurance premiums. Private insurance premiums are not significantly progressive because Kakwani's index was not significantly different from zero at 95% confidence interval, which indicates that the null of proportionality cannot be rejected. Conversely, direct taxes, out-of-pocket payments and contributions to EPF and SOCSO were significantly progressive whilst direct taxes are significantly regressive as shown by a 95% confidence interval. In addition to the Kakwani's index, concentration indices by finance sources for Malaysia are also presented in Table 2. The concentration indices with the largest positive values for direct taxes indicate that direct taxes were the most concentrated on high income groups. Direct taxes were followed by private insurance premiums, out-of-pocket payments, contributions to EPF and SOCSO, and indirect taxes, in order of decreasing value.

**Table 2:** Cumulative Proportion of Health Payments by Consumption Deciles and Kakwani's indices, Malaysia 1998/99

Indices	Consumption	Direct taxes	Indirect taxes	Contributions to EPF and SOCSO	Private insurance premiums	Out-of- pocket payments
**Gini/Concentration index**	0.4051*	0.8003*	0.3273*	0.4862*	0.6985*	0.5094*
*Robust standard error*	*-*	*0.0072*	*0.0642*	*0.0094*	*001735*	*0.0363*
*95% Confidence intervals*	*-*	*0.6745–0.9261*	*0.3089–0.3457*	*0.4435–0.5289*	*0.3585–1.0385*	*0.4383–0.5805*
**Kakwani indices**	-	0.3952*	-0.0779*	0.0811*	**0.2934**	0.1043*
*Robust standard error*	*-*	*0.0620*	*0.0088*	*0.0204*	*0.1726*	*0.0358*
*95% Confidence intervals*		*0.2737–0.5167*	*-0.0607- * *(-0.0951)*	*0.0412–0.1210*	*0.2917–0.2951*	*0.1018–0.1068*

Note: * significantly different from zero at 5%.

### 4.1 Sensitivity Analysis

Four sensitivity analyses were conducted on Kakwani's indices. First, sensitivity analysis on three different incidence assumptions are shown in Table 3, in which Kakwani's index was 0.217, 0.204 and 0.220 for case 1, 2 and 3, respectively. These positive values indicate that the whole health financing system was progressive regardless of the assumption made. The Kakwani's indices for the whole health financing system, with uncertainty intervals (calculated as the deviation from case 1) of merely ± 0.01, are comparatively less sensitive than individual finance sources (with an uncertainty interval of up to ± 0.10 for direct taxes and private insurance premiums).

**Table 3:** Kakwani's Indices under Three Set of Incidence Assumptions on Unallocated Revenue

**Finance sources**	**Kakwani by source**	**Weighted Kakwani's index**			Uncertainty interval
			
		Case 1	Case 2	Case 3	
Direct taxes	0.3952	0.1474	0.1289	0.0723	± 0.10
Indirect taxes	-0.0779	-0.0179	-0.0216	-0.0121	± 0.05
Contributions to EPF and SOCSO	0.0811	0.0004	0.0004	0.0010	± 0.05
Private insurance premiums	0.2934	0.0857	0.0857	0.1420	± 0.10
Out-of-pocket payments	0.1043	0.0010	0.0104	0.0172	± 0.05
**Kakwani's indices for total health finance**	**-**	**0.2166**	**0.2038**	**0.2204**	± 0.01

Second, sensitivity analysis on different equivalence scale and different economies of scale are shown in Table 4. Kakwani's indices were within the range of 0.01 from the base case adopted in this research (adult equivalent scale at 0.5 and economies of scale at 1). The sensitivity analysis shows that the different scales are within the uncertainty interval of ± 0.01 and make little difference to the estimated Kakwani's indices.

**Table 4:** Kakwani's Indices on different adult equivalence scales and different economies of scale

Case	Adult equivalent scale	Economies of scale	Kakwani's indices				
			
			Direct taxes	Indirect taxes	EPF and SOCSO	Private insurance	Out-of-pocket payments
1	0.5	1	0.3952	-0.0779	0.0811	0.2934	0.1043
2	0.5	0.75	0.3951	-0.0766	0.0811	0.2884	0.1034
3	0.5	0.5	0.3825	-0.0750	0.0813	0.2826	0.1021
4	0.4	0.75	0.3950	-0.0778	0.0813	0.2932	0.1042
5	0.3	0.75	0.4001	-0.0789	0.0802	0.2980	0.1047
Range of indices from Case 1			± 0.01	± 0.01	± 0.01	± 0.01	± 0.01

Third, Kakwani's indices were recalculated for three scenarios (of different ATP measures) and shown in Table 5. All three scenarios produce slightly different Kakwani's indices. The progressivity results for the five finance sources remain the same, except out-of-pocket payments (but the change is insignificant). Out-of-pocket payments were found to be mildly regressive when income was used as an ATP measure (instead of mildly progressive when consumption was used in the base scenario). There was no consistent pattern in the changes of Kakwani's index. All Kakwani's indices have an uncertainty interval of ± 0.10 except indirect taxes and out-of-pocket payments (with a greater uncertainty interval of ± 0.15). The choice of ATP measures and the conversion to equivalent adult do have an impact on resultant Kakwani's indices, and should be made explicit while interpreting results and drawing policy implications.

**Table 5:** Concentration Indices and Kakwani's Indices for Sensitivity Analysis on Equivalence Scale and ATP Measures

Description	ATP measures	Direct tax	Indirect tax	Contribution to EPF and SOCSO	Private insurance premium	Out-of- pocket payment
**1. Base Scenario (Payment Per Adult Equivalent on Consumption Measure)**						

Gini/CI *(Robust SE)*	0.4051* (0.0072)	0.8003* (0.0642)	0.3273* (0.0094)	0.4862* (0.0218)	0.6985* (0.1735)	0.5094* (0.0363)
Kakwani's indices *(Robust SE)*	-	0.3952* (0.0620)	-0.0779* (0.0088)	0.0811* (0.0204)	0.2934 (0.1726)	0.1043* (0.0358)

**2. Sensitivity Analysis**						

***i. Per Household Amounts for Payment and Consumption (instead of Per Adult Equivalent Amounts for Payments and Consumption)***						

Gini/CI *(Robust SE)*	0.3839* (0.0066)	0.8055* (0.0628)	0.3102* (0.0077)	0.4540* (0.0191)	0.7007* (0.1654)	0.5088* (0.0342)
Kakwani's indices *(Robust SE)*	-	0.4216* (0.0610)	-0.0737* (0.0079)	0.0700* (0.0188)	0.3168 (0.1646)	0.1249* (0.0341)

***ii. Income Measure instead of Consumption Measure***						

Gini/CI *(Robust SE)*	0.4595* (0.0103)	0.8231* (0.0641)	0.2782* (0.0094)	0.5878* (0.0220)	0.6792* (0.1739)	0.4539* (0.0339)
Kakwani's indices *(Robust SE)*	-	0.3636* (0.0585)	-0.1813* (0.0116)	0.1283* (0.0175)	0.2197 (0.1709)	-0.0056 (0.0341)

***iii. Non Food Consumption Measure instead of Consumption Measure***						

Gini/CI *(Robust SE)*	0.4762* (0.0090)	0.8008* (0.0642)	0.3194* (0.0095)	0.4971* (0.0218)	0.6970* (0.1733)	0.5013* (0.0363)
Kakwani's indices *(Robust SE)*	-	0.3246* (0.0615)	-0.1568* (0.0096)	0.0209 (0.0203)	0.2208 (0.1721)	0.0251 (0.0361)
Uncertainty interval		± 0.15	± 0.10	± 0.10	± 0.10	± 0.15

Note: Gini/CI is Gini coefficient or Concentration index; robust SE is robust standard error.

In the first scenario using actual payments and consumption (without the application of equivalence scale), contributions to EPF and SOCSO were slightly more progressive; indirect taxes were less regressive; whilst the other three finance sources were more progressive. In the second scenario using income as the ATP measure, contributions to EPF and SOCSO were slightly more progressive; indirect taxes were more regressive; and the other three finance sources were less progressive. Nonetheless, out-of-pocket payments have become mildly regressive (instead of progressive in the base scenario). In the third scenario using non food consumption as the measure, indirect taxes were slightly more regressive and the other four finance sources were less progressive.

Fourth, in addition to the base case in Table 3, sensitivity of Kakwani's indices for three scenarios under three different incidence assumptions of unallocated revenue are also reproduced and shown in Table 6. The financing system appears to be progressive for all four scenarios and all three cases, with Kakwani's indices ranging from 0.129 to 0.246. The most progressive system was produced in the scenario of actual payments and consumption. The scenario of income measure and non food consumption measure estimated a slightly less progressive financing system, in which non food consumption produced the least progressive system with the lowest Kakwani index. The sensitivity analysis confirms that the different incidence assumptions yield uncertainty interval between ± 0.01 and ± 0.03, and hence, make little difference to the estimated Kakwani's indices.

**Table 6:** Kakwani's Indices for whole Financing System given Sensitivity Analysis on Three Set of Incidence Assumptions

Sensitivity Analysis	Kakwani's indices			Uncertainty interval
		
	Case 1	Case 2	Case 3	
Payments Per Adult Equivalent on Consumption Measure (Base scenario)	0.2166	0.2038	0.2204	± 0.01
Per Household Amounts instead of Per Adult Equivalent Amounts	0.2456	0.2224	0.2405	± 0.02
Income Measure instead of Consumption Measure	0.1581	0.1325	0.1453	± 0.02
Non Food Consumption Measure instead of Consumption Measure	0.1521	0.1295	0.1462	± 0.03
Differences from choice of measures	± 0.06	± 0.07	± 0.07	-

Note: The largest difference is 0.087 if comparing all cases to Case 1 in Base Scenario, and 0.1161 if comparing all cases among each other.

## 5. Discussion

The Malaysian health financing system was progressive in 1998/99, with a Kakwani index of 0.217. The households contribute progressively towards direct taxes, contributions to social insurance, private insurance premiums and out-of-pocket payments. Indirect taxes emerged as the only regressive finance source. All of the five finance sources were concentrated in the richer groups. The rich make greater payments in relative terms through all five financing mechanisms than the poor, making them progressive in their impact.

The financial burden of the four progressive health payments was concentrated among the higher income groups as compared to proportionality. Direct taxes were the most progressive finance source with the highest concentration level, in which the rich make a much greater proportion of their ATP compared to the poor. The income taxes (which represent direct taxes) schedule was progressive and concentrated in the higher income groups (who are taxed at a higher rate). Private insurance premiums were the second most progressive finance source. The affluent voluntarily purchased private insurance (and chose policy with premiums in accordance with their ATP), in order to protect and safeguard themselves against catastrophic health payments. Out-of-pocket payments were the third progressive finance source. Households' probably adopt selective behaviour while purchasing medical products in a diverse market and seeking health services in a parallel existence of public and private health providers. The switch to private health services by the rich and the predominant reliance on subsidized public health services by the poor resulted in slightly progressive out-of-pocket payments. The fourth progressive source was social insurance contributions which was probably the result of the offsetting effects from the mildly progressive EPF contributions and regressive SOCSO contributions. The EPF contribution schedule appeared to be mildly progressive. Conversely, the imposition of an upper earning limit for the SOCSO contributions and the ineligibility of the affluent as members make SOCSO contributions regressive. The consequences of the ineligibility of the affluent as SOCSO members are regressivity. Thus progressivity nonetheless is captured overall due to the aggregation of EPF and SOCSO contributions by households in the HES 1998/99.

In contrast, indirect taxes emerged to be the only regressive finance source. Indirect taxes were slightly concentrated within the richer groups and were less than proportional. Sales taxes (represented by indirect taxes) are levied depending on the type of goods, irrespective of the households' ATP. The poor have low ATP and end up spending a high proportion of their ATP on purchasing goods. The poor consequently paid more indirect taxes proportionally than the rich.

### 5.1 Methodological Challenges

One of the methodological challenges is to congregate the data from the MNHA and the HES. The original MNHA is employed to establish the macro weights, which are subsequently used to weight the households' contribution towards financing health care services. At one end, MNHA categorized finance sources at the macro (or national) perspective. At the opposite end, the HES collected health expenditure at the micro (or household) level. Inevitably, some MNHA finance sources at the macro level could not be allocated down to the household level in the HES. This difficulty in combining macro and micro level data was exacerbated by the aggregation of the general government revenue. Assumptions therefore had to be made about their distribution in generating macro-weights from the MNHA. Sensitivity analysis nonetheless reveals few differences between three incidence assumptions of their distribution. This seems to suggest that Kakwani's index is an appropriate tool that is capable of producing consistent results within acceptable levels of uncertainty.

The distribution of unallocated finance sources was unknown and their incidence was assumed in establishing the macro weights in this research. The overall taxes in developing countries were in fact found to be broadly progressive by Shah and Whalley [23]. They [23] also found that export duties were progressive; import duties were regressive or proportional; excise duties were generally progressive; value added taxes (which is quite similar to the regressive sales tax in Malaysia) were regressive; whilst company taxes have mixed progressivity (regressive for the lowest income groups, proportional for the middle income groups and progressive for the high income groups) in developing countries.

### 5.2 Comparison with other Malaysian and International Studies

This research assessed equity of health care financing in Malaysia employing Kakwani's progressivity index. It represents the first study to measure progressivity of each of the finance sources and the whole financing system in Malaysia in a comprehensive manner. An alternative methodology, fair financing was measured by the WHO in the World Health Report 2000. With a fairness of financial contribution index of 0.917 (with an uncertainty interval of possible scores between 0.881 to 0.948), Malaysia was ranked at 122–123 from 191 member countries. Subsequently, the MOH Malaysia measured the similar fairness of financial contribution index using the HES Malaysia 1998/99 with complementary data sources and found that the fairness of financial contribution index was 0.982 [24]. It was very close to 1 of perfect equality, which indicates that the financial contribution in Malaysia is fairly and equally distributed. Clearly, the MOH's index was much higher than the 0.917 stated in the World Health Report 2000, which was presumably due to the different data sources used (1980's Malaysian HES versus 1998/99 HES). Additionally, given that the fairness of financial contribution index has been criticised as being unable to distinguish between health financing system that are regressive from those that are progressive [25], it would be beneficial to use Kakwani's index as an alternative index to assess the equity in health care financing in Malaysia. Furthermore, the progressive results from the Kakwani's index demonstrates equitable financing in the Malaysian health financing system, whilst also confirming the applicability of Kakwani's index in another WHO member country.

Apart from Malaysia, five country-specific studies have been reported for Australia [26], Finland [27], Italy [28], the Netherlands [29] and Hungary [30]. Additionally, comparative studies have been carried out in assessing equity in health care financing in 14 OECD countries [31-34], three South Asian countries [35], and 13 Asian countries [22]. All of the Kakwani's indices in 29 countries, including Malaysia, are summarised in Table 7. Countries are grouped into tax financed, social insurance, private insurance or out-of-pocket payments, based on their main finance sources. Tax financed systems were regressive in Denmark, Sweden and Portugal. The regressivity of tax financed systems was attributed to a heavy reliance on the nearly proportional local income tax (in Denmark and Sweden) and a high proportion of regressive out-of-pocket payments (in Portugal). Contradictory progressive results were found for Malaysia, Thailand and Hong Kong. This presumably reflects that these Asian countries have progressive taxes that are channelled to fund government services including public health services, whilst private health services are accessed discriminately by the affluent. The tax financed system was progressive in Malaysia because of its heavy reliance on the general government revenue that mainly consists of progressive direct taxes. A significant contribution from progressive taxes seems to be the prerequisite for any country striving towards equitable tax financing. These Kakwani's indices nonetheless merely serve as brief comparison because of the incomparability in the methodology and data sources (such as years of data) used in studies.

**Table 7:** Kakwani's Indices of Financing Systems in 29 Countries

Countries, Year	Proportion of main finance source (%)	Kakwani's Indices	Progressivity
***Tax financed***			

Malaysia^1^, 1999	60.3	0.2166	Progressive
Thailand, 2000 (O'Donnell et al 2005)	56.3	0.1972	
Hong Kong, 2000 (O'Donnell et al 2005)	55.6	0.1689	

Sri Lanka^2^, 1997 (O'Donnell et al 2005)	49.5	0.0850	Mildly progressive
United Kingdom, 1993 (Wagstaff et al 1999)	64.0	0.0510	
India (Punjab), 1996 (O'Donnell et al 2005)	40.7	0.0485	
Australia, 1989 (Lairson et al 1995)	62.6	0.0100	
Kyrgyzstan, 2000 (O'Donnell et al 2005)	44.5	0.0087	
Finland, 1994 (Wagstaff et al 1999)	75.0	0.0050	
Spain, 1990 (Wagstaff et al 1999)	56.3	0.0004	

Denmark, 1987 (Wagstaff et al 1999)	84.7	-0.0047	Mildly regressive
Sweden, 1990 (Wagstaff et al 1999)	71.9	-0.0158	
Portugal, 1990 (Wagstaff et al 1999)	55.2	-0.0445	

***Social insurance***			

Italy^3^, 1991 (Wagstaff et al 1999)	39.2	0.0413	Mildly progressive
France, 1989 (Wagstaff et al 1999)	73.6	0.0012	

Belgium, 1997 (Wagstaff et al 1999)	42.1	-0.0001	Nearly proportional

Hungary, 1999 (Wagstaff et al 1999)	44.1	-0.0181	Mildly regressive
South Korea, 2000 (O'Donnell et al 2005)	33.9	-0.0239	
Taiwan, 2000 (O'Donnell et al 2005)	51.8	-0.0292	
West Germany, 1989 (O'Donnell et al 2005)	65.0	-0.0452	
Japan, 2001 (O'Donnell et al 2005)	54.0	-0.0688	
Netherlands, 1992 (Wagstaff et al 1999)	64.7	-0.0703	

***Private insurance***			

United States, 1987 (Wagstaff et al 1999)	29.2	-0.1303	Regressive
Switzerland, 1992 (Wagstaff et al 1999)	40.5	-0.1402	

***Out-of-pocket***			

Bangladesh, 1999 (Institute of Policy Studies 2002)	27.2	0.2142	Progressive
Indonesia, 2001 (O'Donnell et al 2005)	33.0	0.1732	
Philippines, 1999 (O'Donnell et al 2005)	39.7	0.1631	

Nepal, 1996 (O'Donnell et al 2005)	23.5	0.0625	Mildly progressive
China, 2000 (O'Donnell et al 2005)	14.9	0.0404	

Note:

^1^The proportion of taxation based on the World Health Report 2006, in which government expenditure constitute 52.9% of the total health expenditure and 99% of this comes from revenue or taxation.

^2 ^Kakwani's index calculated using consumption as the proxy for ability to pay, from the Equitap project.

^3^Italy is grouped under social insurance, based on the highest proportion of finance sources from social insurance.

### 5.3 Policy Implications

The tax-financed system in Malaysia was progressive, indicating that it was equitable. Equitable financing is in line with the vision of a nation moving towards an egalitarian society [36]. The structure of a tax-financed system and a two tier delivery system (a mix of public and private providers) contributes to the equity of health care financing in Malaysia. Households contribute progressively to direct taxes but regressively towards indirect taxes, both of which become general government revenue that are used to fund public health services. All households enjoy public health services that are mostly free, which are heavily relied upon by the vulnerable. Households in active employment benefited from their wages related savings in EPF (that can be reimbursed for medical expenses) while those with low income are protected by additional health plans provided under SOCSO. The affluent conversely incurred out-of-pocket payments, or covered by private insurance plans, while purchasing private health services.

The parallel existence of public and private health services permits the rich to voluntarily switch to the private health services, whilst the poor rely on the public health services. Private health services are seen as a luxury good relative to highly subsidised public health services. While the population suffered the 1997 Asian financial crisis, private hospitals have experienced reduced utilisation, public health services in contrast have experienced increased utilisation of about 15% [6]. The switch of the affluent to private health services is likely to diminish demand in the public sector and reduce government's subsidies for the affluent. Government subsidies could in turn be channelled to the poor who rely on the public health services.

Collaboration from both the public and private sectors was promoted by the government. The private sector is encouraged to deliver appropriate health services to meet population needs, instead of being solely profit-oriented. The government views integration from both public and private sector as important to ensure universal accessibility and comprehensive coverage of health care services [7]. Given that the health care financing is found to be equitable in this research, the private sector could complement the public sector in providing adequate coverage and in facilitating accessibility whilst the public sector could continue to provide health care services through planning and placement of public facilities.

In addition to equity, the efficiency of health care is also closely related to the tax-financed system and the public private provision mix. The efficiency of public health services are monitored by the MOH with performance indicators, whilst the private health services are regulated by Private Healthcare Facilities and Services Act 1998. The public health services, which are almost fully subsidised by the government, place competitive pressure on private health providers to set a reasonable price and to be efficient.

### 5.4 Improving Equity

The progressivity of the whole health financing system depends upon the progressivity of finance sources, which is the distribution of health care costs across deciles within any single finance source. The five finance sources have quite different distributional consequences. Theoretically, the progressivity of a finance source will be improved by shifting the financial burden towards the rich. For instance, a progressive income tax schedule (with lower tax rates on low income groups and higher tax rates on higher income groups) and high sales tax rates on luxury goods place greater financial burden on the rich than the poor. A high tax rate on the high earners nonetheless would not provide incentives for active employment. Correspondingly, high sales tax rates on luxury goods would impede the market performance of luxury goods. Increasing the progressivity of direct taxes or decreasing the regressivity of indirect taxes would, nonetheless, involve revamping the whole tax structure. The progressivity of the EPF schedule will alternatively be improved by a progressive EPF schedule, rather than a uniform flat rate. The regressivity of SOCSO contribution will be reduced by the enrolment of high income groups to the scheme. Notwithstanding, it contradicts with the SOCSO's philosophy of protecting low income employees.

Equity can be improved by changing the financing mix as well as improving the progressivity of the finance sources. Both characteristics are important determinant of the final distribution. Financing strategies with a shift towards increased reliance on the four progressive finance sources and reduced reliance on the regressive indirect taxes (such as the extension of progressive EPF to encompass public employees, the abolishment of upper income limit of SOCSO members, and the promotion of progressive private insurance and out-of-pocket payments as complementary finance sources) might potentially improve equity in health care financing. The equity impact of financing strategies can be monitored by measuring the progressivity over time, in order to provide evidence in implementing health financing policy. Financing strategies that produced an increment in the Kakwani's index of more than 0.10 (in line with its sensitivity within 0.10 provided that the ATP measure is held constant) are considered as of significant impacts and to be recommended for policy consideration.

## 6. Conclusion

Private insurance and out-of-pocket payment are progressive private finance sources in Malaysia. The World Health Report 2006 [8] estimated that the total private finance sources account for 41.8% of total health expenditure in Malaysia. Private finance sources can be seen to constitute a high proportion of total health expenditure in Malaysia. Generally, private financing of health care is seen to be undesirable from an equity point of view. The World Health Report 2000 [9] stated that a high proportion of private finance sources is likely to affect the equity of financing because private health payments might impose disproportionate financial burden on households. Private financing of health care is said to impose high financial burden on high risk groups, predominantly lower income groups, given that the poor tend to have lower health status and higher health risk [9]. Notwithstanding the progressive nature of private finance sources in Malaysia, a growing share of private finance sources without sufficient supports from public funding is likely to undermine equity.

Direct taxes and contributions to EPF and SOCSO are progressive public finance sources. The World Health Report 2000 [9] stated that increased public financing of health care was an integral part of the effort to achieve equity in access. In other words, a growing share of direct tax, indirect taxes and contribution to EPF and SOCSO is seen to contribute to improved equity. This paper found that indirect taxes and contributions to EPF and SOCSO were progressive, indirect taxes were nonetheless regressive. Within public financing, the shifts in favour of progressive income taxes or EPF contribution would therefore lead to a more equitable financing system.

In conclusion, it has been seen that the five finance sources have produced a progressive system that is regarded as equitable. Equity in health care financing was achieved by the predominantly tax financed system with the two tier delivery structure in Malaysia.

## Declaration of Competing interests

The authors declare that they have no competing interests.

## Authors' contributions

CPY conducted the health economics studies, performed the statistical analysis and drafted the manuscript, DKW and THS participated in the design of the study, the development and writing of the manuscript. All authors read and approved the final manuscript.

## Appendix 1

### Estimates of Macro Weights with Three Incidence Assumption

The original macro weights in MNHA are shown in Additional file 1. These macro weights were derived from an unpublished report (Ministry of Health 2005), which contained the disaggregated macro weights required for this research (only accessible for year 2003). Given that Malaysia remains to be tax financing the health care sector without any changes, the role of various sources for health care funds have been maintained within the range of 48% to 61% for public finance sources and 38% to 52% for private finance sources throughout the 5 years (1998–2003).

Three of the five health payments made by the households were matched to the categories of finance sources under the MNHA classification. Out-of-pocket payments were matched to private household out-of-pocket expenditure whilst private insurance premiums were matched to private insurance enterprise (other than social insurance), both as private finance source in the MNHA. Contributions to EPF and SOCSO were matched to EPF and SOCSO, as two distinct public finance sources in the MMHA. Given that contributions to EPF and SOCSO are captured together as a single expenditure in the HES 1998/99, the weights of EPF and SOCSO in the MNHA were combined for analysis. In the MNHA, public finance sources are mainly the MOH, Ministry of Education, local authorities, other federal agencies (including statutory bodies) and Ministry of Defence. Nevertheless, these public finance sources are intermediaries that receive allocation from the Treasury. The Treasury collects taxes from the population and corporations as government revenue, which they use to fund public services.

In this paper, macro weights for the five finance sources were generated based on the MNHA, whereby assumptions had to be made about the distribution of unallocated finance sources. For private finance sources, it was assumed that the three unallocated sources (private corporations, private MCO and NGO) were distributed as a weighted average of the two allocated sources (private household out-of-pocket expenditures and private insurance). This incidence assumption also means that Kakwani's index only measures the progressivity of the two private finance sources which can be allocated down to households.

In terms of public finance sources, most of the general government revenue (financial intermediaries are MOH, Ministry of Education, Local authorities, Federal agencies and Ministry of Defence) are collected as taxes. Nevertheless, income tax and sales tax are the only two taxes that can be allocated down to the households. The proportion of direct taxes (represented by income taxes) and indirect taxes (represented by sales taxes) is estimated from the Eighth Malaysia Plan. The distribution of some taxes (for example company taxes, petroleum taxes, export duties, import duties and excise duties) is difficult to estimate from the HES data. The same is true for non-tax revenue. Assumptions were made about the distribution of unallocated tax burden. The distribution of allocated taxes (such as income taxes and sale taxes) is used to reflect the distribution of unallocated taxes in some related way by making different incidence assumptions. Macro weights with different incidence assumptions are conducted by examining these three cases.

Additional file 2 shows the proportion of general government revenue from the Eighth Malaysia Plan and the weights of finance sources under three set of incidence assumptions. In Case 1, it is assumed that direct taxes are distributed as income taxes, indirect taxes are distributed as sales taxes, and the remainder of unallocated revenues are distributed as a weighted average of allocated taxes. In Case 2, it is assumed that unallocated revenues are distributed as the weighted average of allocated taxes. In Case 3, it is assumed that unallocated revenues are distributed as a weighted average of all allocated payments, which also means that Kakwani's indices only measure the progressivity of those finance sources which can be allocated down to households.

The macro weights for these three cases were created for the sole purpose of calculating Kakwani's indices and therefore do not represent the exact weights of finance sources in the country. The incidence assumption in Case 1 was used as base-case analysis, whilst incidence assumptions of Case 2 and Case 3 were used as sensitivity analysis to test how Kakwani's indices change when different incidence assumptions are made on the distribution of unallocated revenues.

## Supplementary Material

Additional file 1 Macro weights in MNHA. The table shows the estimates of macro weights that are derived from the MNHA. Click here for file

Additional file 2 Estimates of Macro Weights with Three Incidence Assumptions. The table shows the proportion of general government revenue from the Eighth Malaysia Plan and the weights of finance sources under three set of incidence assumptions. Click here for file
