# Supplementary material for: Equity in health care financing: The case of Malaysia
Source: Int J Equity Health. 2008 Jun 9;7:15. doi: 10.1186/1475-9276-7-15 (PMC2467419; doi:10.1186/1475-9276-7-15)
Supplement: Additional file 1 — Macro weights in MNHA. The table shows the estimates of macro weights that are derived from the MNHA. [file 1475-9276-7-15-S1.doc]

Additional file 1: Macro weights in MNHA

| Malaysian National Health Accounts | Amount (RM million) | Weights (%) | Health Payment |
| --- | --- | --- | --- |
| **Public Finance Sources** | **7,593** | **60.83** |  |
| Ministry of Health | 6,511 | 52.16 |  |
| Ministry of Education | 481 | 3.85 |  |
| Local authorities | 344 | 2.75 |  |
| Other federal agencies (including statutory bodies) | 162 | 1.30 |  |
| *Employee Provident Funds (EPF)* | *37* | *0.30* | *EPF Contribution* |
| Ministry of Defence (MOD) | 36 | 0.29 |  |
| *Social Security Organization (SOCSO)* | *22* | *0.18* | *SOCSO Contribution* |
| **Private Finance Sources** | **4,890** | **39.17** |  |
| *Private household out-of-pocket expenditures* | *2,261* | *18.11* | *Out-of-pocket payment* |
| All corporations (other than health insurance) | 1,746 | 13.99 | *-* |
| *Private insurance enterprises (other than social insurance)* | *769* | *6.16* | *Private insurance* |
| Private MCO and other similar entities | 75 | 0.60 | - |
| Non-profit organizations serving households | 39 | 0.31 | - |
| **Total** | **12,483** | **100** | **-** |

Note: Estimates for the macro weights from the MNHA are found to be different from the estimates of WHO. The weights of the WHO are: public finance sources at 58.2%, private finance sources at 41.8% (in which 30.8% is out-of-pocket payments and 5.7% is private insurance).

[Source: Ministry of Health 2005]
