# Supplementary material for: Equity in health care financing: The case of Malaysia
Source: Int J Equity Health. 2008 Jun 9;7:15. doi: 10.1186/1475-9276-7-15 (PMC2467419; doi:10.1186/1475-9276-7-15)
Supplement: Additional file 2 — Estimates of Macro Weights with Three Incidence Assumptions. The table shows the proportion of general government revenue from the Eighth Malaysia Plan and the weights of finance sources under three set of incidence assumptions. [file 1475-9276-7-15-S2.doc]

Additional file 2: Estimates of Macro Weights with Three Incidence Assumptions

| **Finance source** | Amount  (RM million) | Proportion (%) | Case 1 | Case 2 | Case 3 |
| --- | --- | --- | --- | --- | --- |
| ***A. Public Finance Sources*** |  | ***60.83*** |  |  |  |
| General government revenues | - | 60.35 | - | - | - |
| *Direct taxes* | 29156 | 28.45 | - | - | - |
| Income taxes | 27016 | 26.36 | - | - | - |
| Company | 13905 | 13.56 | - | - | - |
| **Individual** | **7015** | **6.84** | **0.38** | **0.33** | **0.19** |
| Petroleum | 6010 | 5.86 | - | - | - |
| Other direct taxes | 2410 | 2.35 | - | - | - |
| *Indirect taxes* | 18017 | 17.58 | - | - | - |
| Export duties | 1032 | 1.01 | - | - | - |
| Petroleum | 999 | 0.97 | - | - | - |
| Palm oil | 4 | 0.00 | - | - | - |
| Others | 29 | 0.03 | - | - | - |
| Import duties | 3599 | 3.51 | - | - | - |
| Excise duties | 3803 | 3.71 | - | - | - |
| **Sales tax** | **5968** | **5.82** | **0.23** | **0.28** | **0.16** |
| Service tax | 1701 | 1.66 | - | - | - |
| Other indirect taxes | 1914 | 1.87 | - | - | - |
| *Non-tax revenue* | 14097 | 13.75 | - | - | - |
| Petroleum | 6384 | 6.23 | - | - | - |
| Other non-tax revenue | 7713 | 7.52 | - | - | - |
| *Non-revenue receipts* | 594 | 0.58 | - | - | - |
| **Contributions to EPF and SOCSO** | **-** | **0.48** | **0.00** | **0.00** | **0.01** |
| ***B. Private Finance Sources*** |  | ***39.17*** |  |  |  |
| **Out-of-pocket payment** | **-** | **18.11** | **0.29** | **0.29** | **0.48** |
| All corporations (other than health insurance) | - | 13.99 | - | - | - |
| **Private insurance** | **-** | **6.16** | **0.10** | **0.10** | **0.16** |
| Private MCO and other similar entities | - | 0.60 | - | - | - |
| Non profit organization serving households | - | 0.31 | - | - | - |
| ***Total*** |  | ***100.00*** | ***1.00*** | ***1.00*** | ***1.00*** |

[Source: Economic Planning Unit 2001

and Ministry of Health 2005]
